# An Exploration of Computer Game-Based Instruction in the “World History” Class in Secondary Education: A Comparative Study in China

**DOI:** 10.1371/journal.pone.0096865

**Published:** 2014-05-09

**Authors:** Zhonggen Yu, Wei Hua Yu, Xiaohui Fan, Xiao Wang

**Affiliations:** 1 School of Foreign Languages, Hohai University, Nanjing, China; 2 School of English, Zhejiang Yuexiu University of Foreign Languages, Shaoxing, China; University of Westminster, United Kingdom

## Abstract

So far, many studies on educational games have been carried out in America and Europe. Very few related empirical studies, however, have been conducted in China. This study, combining both quantitative with qualitative research methods, possibly compensated for this regret. The study compared data collected from two randomly selected classes (out of 13 classes) under computer game-based instruction (CGBI) and non-computer game-based instruction (NCGBI), respectively, in a senior high school located in Nanjing, Capital of Jiangsu Province, in China. The participants were 103 students, composed of 52 boys and 51 girls (aged 17-18 years old). The following conclusion was reached: (1) participants under CGBI obtained significantly greater learning achievement than those under NCGBI; (2) participants were significantly more motivated by CGBI compared with NCGBI; (3) there were no significant differences in learning achievement between boys and girls; although (4) boys were significantly more motivated by CGBI than girls. Both disadvantages and advantages were discussed, together with directions for future research.

## Introduction

History is full of changes and rich in facts. The integration of historic facts into education may bring recreation and relaxation to education, thus stimulating learning interest and improving learning achievements [1].

With increasing interest in effectiveness of computer games in education, a growing number of scholars are exploring game-based learning in history. A substantial body of studies revealed that computer games could establish powerful learning environments [2]–[7], but critics of gaming have questioned the value of what students actually learn in these environments as well.

In China, it is a must for senior high students to enroll for the curriculum “World History”, which tends to be taught through traditional multimedia and blackboard. Very few studies have been conducted on the effectiveness of computer game-based instruction (CGBI) in the sphere of world history. Furthermore, a growing number of girls are using games [8]. Further studies on gender differences in the motivational impact and the learning achievement of CGBI are needed because empirical studies have yielded contradictory findings [9]. This study, contrasting most studies conducted in Europe and America [7], [9], [10], is carried out in China. Studies on game-based instruction in China are sparse so it is considered necessary and meaningful.

This study, aiming to identify the effectiveness of CGBI, attempts to answer two questions: (1) Will CGBI significantly stimulate motivation and improve learning achievement compared with non computer game-based instruction (NCGBI)? (2) Will boys obtain significantly more learning achievement and be more motivated than girls in the CGBI class?

## Literature Review

### CGBI and learning achievement

It has long been suggested that computer games might be useful in formal educative contexts [11]. Since 1985, computer games and their impact on education, and especially on students and teachers, have advanced in leaps and bounds. The effectiveness of computer games in class was confirmed in the aspects of learning, motivation and classroom dynamics [12].

CGBI was considered as a beneficial educational approach, as games could make learning interesting, endow students with independence, wide scope of knowledge and quality of enquiry [13], enhanced cognitive abilities supporting one's overall ability to learn [14], and could facilitate task switching and perceptual speed [15]–[17]. Additionally, computer games were able to motivate learners [18], due to their intrinsically motivating character [19]–[22].

The question of whether and under what circumstances games are effective still remains open. A number of review articles and meta-analyses focused on the general effectiveness of serious, video, simulation, and computer games for learning. After comprehensive review, it was found that computer games could have both positive and negative impacts on education [23], and it seems impossible to reach any reliable conclusion concerning the effectiveness of serious games in education [24]. Some evidence was found for the effects of video games on language learning, history, and physical education (specifically exergames), but little support was revealed for the academic value of video games in science and math after reviewing many articles [25]. Nevertheless, simulation games as a supplement to other instructional methods were proved effective in education [26].

### CGBI and motivation

The majority of youngsters enjoyed computer games but disliked the boring contents in textbooks [8]. The features of computer games were believed to have shaped students' cognitive abilities of and motivation towards learning, contrasting with monotonous and unattractive scholastic contents in traditional instruction [27]. There was a sharp contrast between formal education and computer games [28]. Nevertheless, motivation towards computer games could integrate dull curricula into what Prensky [8] referred to as ‘Digital Game-Based Learning’ (DGBL). Games used in teaching and learning were thought to be able to make school work more interesting, more easily understood and thus more effective [29]–[31], due to which learners tended to be positive towards CGBI. The motivational potentialities of computer games have led to considerable interest in their use in educational contexts [32]–[34].

### CGBI and gender differences

A disputable issue is whether CGBI is equally motivating and effective for both genders. Games, especially those comprising combats, were subject to male domination [35]. Previous literature showed that boys tended to play computer games more frequently, intensively and skillfully than girls [36]–[38]. Unsurprisingly, boys developed greater familiarity with computer games and greater computer belief and capability than girls [9], [39]. Therefore, it seems reasonable to assume that CGBI may be a better choice for boys compared with girls and boys may be more motivated by CGBI than girls.

However, studies on gender differences revealed various scenarios. The study conducted by Young and Upitis [40], also discussed in De Jean, Upitis, Koch, and Young [41], revealed that more females than males showed motivation towards the game due to their appreciation of the female protagonist, while more boys than girls showed more motivation towards the game goal, mastered productive skills to share information, and were better in perceiving mathematics. It was also discussed that different genders preferred different games and males played computer games longer than females [42].

Boys and girls also showed different preferred games, and different levels of performance. Boys made faster progress, although no significant gender differences were revealed in terms of learning achievement [43]. Despite boys' greater involvement with, liking of and experience in computer gaming, and their greater initial computer memory knowledge, the learning gains that boys and girls achieved through the use of the game, however, did not differ significantly, and the game was found to be equally positively evaluated for both boys and girls. Educational computer games could be exploited as effective and motivational learning environments, regardless of students' gender [9].

However, the question should not be whether gender influences the appropriateness or effectiveness of games for learning, but instead should be how stereotypical views of designers and even game players themselves influence the design and effectiveness or appropriateness of these games. Long debate still focused on “whether girls do and can and should play video games and women were still vastly underrepresented in the fields that design digital technology” [44]. Video games were designed especially for women and girls through an extremely stereotyped conception of differences between male and female game players and designers produced games catering for female needs. Despite girls and women possibly were less active in gaming than boys and men, it did not mean they were not playing [45]. They might be playing with their male partners [46].

## Methods

The research lacks consent because the data were analyzed anonymously. The research has been approved by the authors' institutional review board-School of Foreign Languages of Hohai University, which waived the need for written informed consent from the participants.

The study used both quantitative and qualitative research methods by comparing the learning achievement and motivational dimensions of both classes, coupled with gender differences in CGBI and NCGBI.

### Participants

The study was conducted between two randomly selected classes (totally 13 classes) in a senior high school located in Nanjing, capital of Jiangsu Province in China. The participants were 103 students, composed of 52 boys and 51 girls, ranging from 17–18 years old [mean (M)  =  17.54, standard deviation (SD)  =  0.50]. The participants were in the second grade and had learned the same subject related to world history based on the teaching outline designed by Ministry of Education of China. No educational software had been used at school until the study was conducted. One class (Class A) was instructed via CGBI, while the other (Class B) via NCGBI. Class A received formal training of how to play the game “The Age of Empires: The Conquerors Expansion”.

The training session lasted for six hours within two days. In the training, researchers introduced the gaming goal to participants and then helped them practice gaming. The researchers guided participants how to install, start, and play the game. After the training, researchers ensured that Class A mastered the gaming skills.

A pretest/posttest experiment was designed in this study in order to explore the effects of two types of instruction (CGBI/NCGBI) on participants' achievement. Furthermore, after the completion of the interventions, participants' motivation towards both instructions was elicited through a feedback questionnaire. Participants' gender was also considered an important factor influencing the data obtained.

### Instruments

To fulfill the goal of the study, one commercial game “The Age of Empires: The Conquerors Expansion” was purchased and applied. Furthermore, three paper-based questionnaires were constructed: (1) a pretest and posttest questionnaire containing two parts (biographical information, world history knowledge test) respectively, and (2) a posttest feedback questionnaire. Data elicited through the instruments would be publicly available (please see Dataset S1 and Dataset S2).

### “The Age of Empires: The Conquerors Expansion”

The computer game “The Age of Empires: The Conquerors Expansion” was developed by Ensemble Studios Corp. for Microsoft Corporation. The Conquerors Expansion was designed to follow the predecessor “The Age of Empires” by including medieval fights and empire construction with many exciting gaming features. There are six levels of difficulty for the game goal, i.e. *the easiest, easy, moderate, difficult, more difficult* and *the most difficult*. In order to win the game at the *moderate* level, the participants were required to command economic and military technologies of different civilizations such as Aztecs, Huns, Koreans, Mayans, and Spanish, coupled with combat rules and units, e.g. Conquistadors, Eagle Warriors, Halberdiers, Hussars, Jaguar Warriors, Missionaries, Petards, Plumed Archers, Tarkans, Turtle Ships, and War Wagons. Military and economic technologies were considered quintessential factors to win the game, which were of various varieties, including Bloodlines, Caravan, Herbal Medicine, Heresy, Parthian Tactics, Theocracy, and Thumb Ring, etc. In order to improve its unique unit or team bonus, each civilization can create a unique technology which cannot be developed by other civilizations.

### The pretest and posttest questionnaires

Questions in both questionnaires were the same except that the ordering of knowledge test on world history was different in order to avoid interference of the remaining memory. The questionnaire was composed of two sections: biographical information and world history knowledge test. The biographical variables elicited the following information: participants' gender, age, school grade, frequency of computer gaming on campus, frequency of computer gaming outside school, liking of computer games, and computer gaming experience. In particular, students were asked to note their gender and age as well as their average school grade in the previous scholastic year [9]. Questions in biographical variables are listed in Table 1.

**Table 1:** Questions in both pretest and posttest questionnaires.

Questions	1	2	3	4	5
How often do you play computer games on campus/outside school?	never	several times per year	Several times per month	several times per week	Several times per day
Do you like playing computer games?/Do you have any experience in playing computer games?	Not at all	A little	Quite a lot	A lot	Very much

As described in Table 1, via two questions, participants were required to answer questions based on a 5-point scale (1  =  ‘never’, 2  =  ‘several times per year’, 3  =  ‘several times per month’, 4  =  ‘several times per week’, 5  =  ‘several times per day’) how often: (a) they played computer games on campus, and (b) they played computer games outside the campus. In the end, they were also asked to answer questions based on a 5-point scale (1  =  ‘not at all’, 2  =  ‘a little’, 3  =  ‘quite a lot’, 4  =  ‘a lot’, 5  =  ‘very much’) their: (a) liking of computer games, (b) experience in computer gaming.

Section 2 of the questionnaire aimed to evaluate participants’ world history knowledge at the initial stage of the study. It was composed of a World History Test, which was designed by the researchers, piloted and reviewed by a panel of high school teachers with rich experience in World History teaching and researching. The test was made up of 30 multiple-choice questions on world history such as the unique military technology in some nations and the historic events during some periods. All the informational content related to those questions was contained in the learning materials of The Age of Empires: The Conquerors Expansion. The questions fundamentally kept consistent with the learning materials.

### The posttest feedback questionnaire

The posttest feedback questionnaire included two parts. The first part aimed to identify the attributes of both CGBI and NCGBI, containing 9 items that involved three motivational dimensions [9]: (a) overall appeal, (b) availability of learning materials, and (c) educational value. Specifically, through 9 closed questions, participants were required to rate on a 5-point scale (1  =  ‘not at all’, 2  =  ‘a little’, 3  =  ‘quite a lot’, 4  =  ‘a lot’, 5  =  ‘very much’) the degree to which they found either CGBI or NCGBI: (1) was interesting, (2) was enjoyable, (3) was engaging, (4) was simple to use, (5) was easy to navigate, (6) comprised satisfactory graphics, (7) comprised subject matter easy to understand, (8) comprised questions easy to answer, (9) was conducive to world history learning. Items 1–3 intended to identify the motivational dimension ‘overall appeal’. The motivational dimension ‘availability of learning materials’ was explored through Items 4–6. The rest 7–9 items aimed to explore the last dimension ‘educational value’.

The second part, via open-ended questions, attempted to elicit participants’ generic view on use of CGBI as well as the proposals for future CGBI. The data elicited through this part was qualitative rather than quantitative elicited through the first part.

### Procedures

Participants in both classes were instructed by the same instructor via either CGBI or NCGBI. Prior to the instruction, participants were required to fill out the pre test questionnaire. After the instruction, which took two hours a week (16 weeks in total) within one semester, the participants were required to finish both posttest questionnaire and posttest feedback questionnaire.

The study compared two modes of instruction on world history knowledge. Both instructions remained the same in terms of teaching outline, learning objectives, material, and contents. The only difference was that one was computer game-based, whereas the other was not. Any differences in learning achievement and motivational dimensions between the two instruction methods, therefore, might be attributed to use or nonuse of the computer game. However, there were still many other variables that may influence the results, such as previous experiences with games for learning, students’ attitudes, actual enjoyment and efforts [47], motivation for participation in the game and instructional quality of the involved teacher [48].

### CGBI

Age of empires: The Conquerors Expansion encourages blended learning where students have access to not only history learning but also game playing. While navigating in the gaming settings, participants were able to identify information, to solve problems, to ponder over the concepts in the learning materials and to sharpen their understandings of historical events. After class, participants were required to complete the assignment allotted by the instructor. Participants' performances were also recorded and a final report was provided as well at the end of the semester.

It was essential for participants to be familiar with historic elements of different nations including military, agriculture, industry, economy, religion and technologies. These elements were extremely important if participants desired to reach the gaming goal. They should know the different advantages of one specific nation over the enemy. They also needed to know the special features of one nation, so that they could make their nation more powerful than the counterpart. For example, they could use the special military or agricultural technique to defeat the enemy or gather the food.

Since all of these elements were important to reach the gaming goal, researchers gave a detailed account to each of them. Then participants started to play the game. They were encouraged to apply the elements of different nations to game playing. They were also required to preview before gaming and review after gaming. Assignment for participants was mainly finished in class, i.e. in the process of gaming. Actually, the goal-reaching process was no less than a world knowledge acquisition class.

This game possibly helped participants to acquire world knowledge better than sitting in a world history class if the game could outweigh classroom learning in terms of interest and efficiency. Participants might feel more interested in gaming than lecturing and thus learned history more efficiently. The game consisted of the whole package of history knowledge. When playing, with intense interest, participants might be gradually familiar with the history knowledge. Urged by the thirst for victory, participants might highly concentrate on gaming. This could hardly be realized in a traditional class. Participants tend to feel dizzy and bored when facing the stark blackboard. Therefore, a solid foundation was paved for this study to compare gaming and lecturing in learning world history.

In order to promote the blended learning with game playing, six elements were considered and adopted [8], [21]: (a) rules to govern learning with computer games, (b) definite goals, (c) catalysts activating participation, (d) levels of achieving goals, (e) control over participation, (f) unknown results, and (g) feedback in time. The game was easily learned and played and thus participants were ready to join. With a view to minimizing gender bias and attracting both boys and girls, the game was oriented to neutral rather than gender-specific. The drawbacks such as violence, indulgence or pornography oriented content were minimized.

In the process of instruction, some problems were encountered. The game was initially set at a “difficult” level, which made the game last more than two hours, exceeding the restricted teaching hour (two hours a week). Later, the gaming difficulty was adjusted and the period was limited to one hour. Furthermore, some participants were still not familiar with the game after training, which also led to time-consuming operation. Consequently, participants were encouraged to acquaint themselves with the game before and after classes. Good players were encouraged to help those unfamiliar with the gaming and the instructor also taught the participants to play the game when necessary.

### NCGBI

NCGBI, whose teaching outline, learning objectives, learning materials, and learning contents were identical to CGBI, was carried out in the classroom by the same instructor who also operated CGBI. Participants learned world history knowledge through a traditional mode. They sat in the classroom, and the teacher delivered the knowledge through a blackboard, chalk, and a multimedia projector system. Participants were required to respond to teacher's questions in class and to finish assignment after class. They were also required to preview what would be learned before class. After class, they also needed to review what had been taught and to finish homework. The teacher kept track of participants' performance in class and gave them a final report at the end of the semester.

The lecturing was conducted two hours a week (16 weeks in total) in the classroom, where the instructor presented the learning materials, history knowledge, and other related points either on the blackboard or on the computer screen projected by the multimedia. The instructor explained the contents and interacted with the participants in the class. Participants could also ask the instructor questions and interact with each other. After class, the instructor assigned tasks for participants to complete at home, with the same procedures as in CGBI.

## Results

The quantitative data were all entered into SPSS and analyzed through corresponding programs, while the qualitative data, i.e. the data elicited from open-ended questions to study participants' generic view on use of CGBI as well as the proposals for future CGBI, were analyzed through summarizing and reasoning.

### The biographic data

The biographic data including frequency of gaming on campus and out of school, liking of computer gaming and experience in computer gaming were entered into SPSS and analyzed by Paired Samples Test comparison, whose results were shown in Table 2.

**Table 2:** Comparison of Biographic Data between Class A and B.

Items	Mean		Paired differences		t	Sig.(2-tailed)
	Class A	Class B	Mean	S.D.		
FGC	1.75	1.78	−.03	.90	−.33	.74
FGOS	2.78	2.72	.06	1.09	.55	.59
LG	3.96	3.91	.05	.92	.54	.59
EG	3.50	3.51	−.01	.92	−.11	.91

Notes: FGC: frequency of gaming on campus; FGOS: frequency of gaming out of school;LG: liking of gaming; EG: experience in computer gaming.

As described in Table 2, the biographic data between Class A (CGBI) and Class B (NCGBI) were compared through the program Paired Samples Test Comparison. It was clearly revealed that participants in both classes failed to show any significant differences in terms of frequency of gaming on campus (*t* = −.33, *p* = .74), frequency of gaming out of school (*t* = .55, *p* = .59), liking of gaming (*t* = .54, *p* = .59), and experience in computer gaming (*t* = −.11, *p* = .91) at the significant level .05. Therefore, it was concluded that the randomly selected participants were statistically homogeneous in terms of the identified biographic data, which formed a valid foundation for further comparative study.

Based on the two research questions mentioned in Section One, the paper intends to confirm the following four hypothyesis.

### Hypothesis 1: Learning achievement in Class A is significantly greater than in Class B

In order to identify whether there were significant differences in learning achievement between CGBI and NCGBI, it was necessary to firstly study the differences in pretests and secondly identify the differences in posttests between both classes regarding world history knowledge, which was shown in Table 3.

**Table 3:** Comparison of learning achievement between Class A and B.

Items	Mean	Paired differences		t	Sig.(2-tailed)
		Mean	S.D.		
PreCGBI	13.53	.32	5.77	.51	.61
PreNCGBI	13.21				
PostCGBI	18.22	1.29	6.20	2.01	.05
PostNCGBI	16.92				

Notes: PreCGBI/NCGBI = pretest of CGBI/NCGBI; PostCGBI/NCGBI = posttest of CGBI/NCGBI.

As shown in Table 3, data between pretests and posttests in terms of CGBI and NCGBI were compared and analyzed. It was clearly shown that, at the significance level .*05*, the mean score of pretest of CGBI (M = 13.53) was not significantly (*t* = .51, *p* = .61) different from that of posttest in NCGBI (M = 13.21), which indicated that both classes were at the same level of world knowledge due to successful random selection. The mean score of posttest in CGBI (M = 18.22), however, was significantly (*p* = .05) higher than that in NCGBI (M = 16.92) at the significance level .*05*. This demonstrated that CGBI produced significantly greater learning achievement than NCGBI. Therefore, the hypothesis “Learning achievement in Class A is significantly greater than in Class B” was confirmed.

### Hypothesis 2: Class A is significantly more motivated compared with Class B

In order to explore different motivations towards both CGBI and NCGBI, data gathered from three motivational dimensions, i.e. overall appellation, availability of learning materials, and educational value, were entered into SPSS and paired samples comparisons were conducted with the result summarized in Table 4.

**Table 4:** Comparison between three motivational dimensions of both CGBI and NCGBI.

Items	Mean		Paired differences		t	Sig.(2-tailed)
			Mean	S.D.		
appealCGBI- appealNCGBI	7.42	7.18	.24	2.29	1.03	.31
availCGBI- availNCGBI	7.71	6.83	.87	2.31	3.69	.00
eduvalCGBI-eduvalNCGBI	7.29	7.31	−.02	2.83	−.07	.94
motCGBI- motNCGBI	22.34	21.37	.97	4.58	2.05	.04

Notes: appealCGBI/NCGBI  =  appellation of CGBI/NCGBI; availCGBI/NCGBI  =  availability of learning materials of CGBI/NCGBI; eduvalCGBI/NCGBI  =  educational value of CGBI/NCGBI; motCGBI/NCGBI  =  motivation of CGBI/NCGBI.

As summarized in Table 4 three motivational dimensions were analyzed and compared between CGBI and NCGBI. It was obvious that appellation in CGBI (M = 7.42) was insignificantly (*p* = .31) different from that in NCGBI (M = 7.18) at the significance level .*05*, so was the dimension “educational value” (*p* = .94). Nevertheless, the dimension “availability of learning materials” in CGBI (M = 7.71) was significantly (*p* = .00) greater than that in NCGBI (M = 6.83) at the significance level .*05*. The general motivation towards CGBI (M = 22.34) also showed a significant (*p* = .04) increase compared with NCGBI (M = 21.37) at the significance level .*05*. This indicated that participants were generally significantly more motivated by CGBI than by NCGBI although there were some insignificant differences in dimensions appellation and educational value. Hence the hypothesis “Class A forms significantly more motivation compared with Class B” was confirmed.

### Hypothesis 3: In Class A, boys exhibit significantly greater learning achievement than girls, whereas the difference is not significant in Class B

To test this hypothesis, it is a must to conduct independent samples T tests on posttests of world history knowledge between boys and girls in both classes. The results were shown in Table 5.

**Table 5:** Comparison of learning achievement between boys and girls in both classes.

Items	Mean		Paired differences		t	Sig.(2-tailed)
	Female	Male	Mean	S.D.		
postCGBI	17.53	18.98	3.22	−1.45	−1.48	.14
postNCGBI	17.45	16.68	3.65	.77	1.01	.31

Note: postCGBI/NCGBI = posttest of CGBI/NCGBI.

As displayed in Table 5, in Class A, girls (M = 17.53) received slightly (*p* = .14) less learning achievement than boys (M = 18.98). In class B, girls (M = 17.45) slightly (*p* = .31) surpassed boys (M = 16.68) in terms of learning achievement. This did not exhibit any significant differences in learning achievement between different genders in both classes at the significance level .*05*. As a result, the hypothesis “In Class A, boys exhibit significantly greater learning achievement than girls, whereas the difference is not significant in Class B” was rejected.

### Hypothesis 4: In Class A, boys are significantly more motivated by CGBI than girls, whereas the difference is not significant in Class B

To identify the differences in motivation towards CGBI between boys and girls, it is necessary to compare their evaluation on CGBI between both classes.

As shown in Table 6, in Class A, males (M = 24.15) were significantly (*p* = .04) more motivated by CGBI than girls (M = 20.79), so was in Class B (*p* = .002) at the significance level .*05*. This indicated that either in Class A or Class B, boys were significantly more motivated by CGBI than girls. Consequently, the hypothesis “In Class A, boys are significantly more motivated by CGBI than girls, whereas the difference is not significant in Class B” was partially confirmed.

**Table 6:** Comparison of motivation between boys and girls in both classes.

Items	Mean		Paired differences		t	Sig.(2-tailed)
	Male	Female	Mean	S.D.		
motCGBI	24.15	20.79	3.35	.63	5.32	.04
motNCGBI	22.85	19.98	2.87	.82	3.48	.002

Note: motCGBI/NCGBI = motivation of CGBI/NCGBI.

### Open-ended questions: what do you think of use of CGBI in education and what proposals do you have for future CGBI?

The researcher enquired the participants about their opinions on the use of CGBI in education and the feedbacks were obtained on the spot. The game “The Age of Empires: The Conquerors Expansion” was firstly made clear and the target subject “World History” was secondly perceived by the students. Many female students had never heard of it and very few of them once played it. Instead they said that they preferred small-scale games to large-scale ones because the large ones were always characteristic of violence, attack, and thrillingness. But what they pursued was gentleness, peacefulness and beauty. But when asked if the game could be applied to education, they felt curious, appeared interested and explored further with the researcher. They nearly universally agreed that if games could be applied to education, education would not be tedious any more, and their learning achievement would most likely be improved. They eagerly longed for future learning via gaming.

By contrast, most male students were familiar with this game and many of them had the experience of playing it. When asked about the opinion on the use in education, most of them felt excited and could not wait for gaming to learn world history. They said they liked this kind of game very much and were aware that this game was helpful to memorize historical events, sites and characters, hence a wonderful tool in education. What they were most concerned about was whether there were any more games able to be used in education, especially in the subjects difficult to make progress, such as mathematics and English. They eagerly hoped for future trial of gaming in education and also suggested some games possibly useful for education.

## Discussion

The findings in this study were generally consistent with those revealed by Papastergiou [9]. In most schools, modern educational technologies may be multimedia projector, language lab, QQ, email, and distant video conference, etc. It is most normal for participants to have expressed their curiosity in gaming to learn although prominent differences exist between males and females. Therefore, participants might have been greatly motivated when invited to game for learning. Much motivated, it is unsurprising that participants in Class A obtained significantly better academic achievement than those in Class B. Males' more familiarity with the game led to the findings that they were significantly more motivated by use of gaming in education than females. Participants in Class A who went through educational gaming were more motivated than those who did not, which proved that CGBI in world history was generally more appealing and interesting at least compared with traditional NCGBI.

On the other hand, some surprising data were also discovered. It is reasonable that more motivated learners should have obtained greater learning outcomes than those less motivated. Males under CGBI, more motivated than females, should have achieved learning objectives in world history knowledge significantly better than females under CGBI. Nevertheless, no significant differences were found in learning achievement between males and females. Females may have felt less interested in and less motivated by CGBI than males, resulting in their less reliance on CGBI. They may, however, have relied on other learning strategies, such as self-education, careful reviewing the contents, listening attentively to the instructor. Males, although motivated by CGBI, may have overlooked other learning methods. Therefore, the learning achievement may have struck a dynamic balance, producing relatively similar academic results. Although the finding was not in conformity with some previous literature [40]–[41] which argued that computer games were more effective with boys than with girls, there was still some literature meeting this finding [8], [49] which contended that genders did not influence the learning achievement despite the fact that computer games were more motivating and appealing to boys than to girls.

## Conclusion

Although numerous studies on educational games have been conducted in the western countries, very few have been done in the mainland Chinese context. This study, carried out in mainland China, may have been meaningful. The conclusion was reached that participants under CGBI obtained significantly greater learning achievement than those under NCGBI; participants were significantly more motivated towards CGBI compared with NCGBI; there were no significant differences in learning achievement between boys and girls; and boys were significantly more motivated by CGBI than girls. There are still various disputes regarding gender differences in CGBI.

This study has some defects. For instance, the same teacher taught both classes using different teaching styles and aprroaches, one in CGBI and the other in NCGBI. It may be hard for the teacher to switch between different styles and approaches from time to time. He may have mixed both styles and approaches sometimes, which may have brought about confusion of the results. In addition, the questionnaires should have gone through further validation and the reliability should have been seriously tested although they were designed by experienced instructors.

In the CGBI field there are still innumerable issues unsolved [50]. Different games may have different influences upon education. One game may be effective in one subject, while the other may not. One game may be effective in America and Europe, but may not be so in Asia. More studies on CGBI are still needed and more appropriate games are awaiting development and application, both of which may be in need of multi-cooperation and cross-disciplinary study.

## Supporting Information

Dataset S1 (ZIP)

Dataset S2 (DOC)
