# Supplementary material for: An Exploration of Computer Game-Based Instruction in the “World History” Class in Secondary Education: A Comparative Study in China
Source: PLoS One. 2014 May 9;9(5):e96865. doi: 10.1371/journal.pone.0096865 (PMC4016116; doi:10.1371/journal.pone.0096865)
Supplement: Dataset S2 — (DOC) [file pone.0096865.s002.doc]

1. age
2. gender: 1=female; 2=male
3. FGCA: How often do you play computer games on campus? CLASS A
4. FGOSA: How often do you play computer games outside school? CLASS A
5. LGA: Do you like playing computer games? CLASS A
6. EGA: Do you have any experience in playing computer games? CLASS A
7. FGCB: How often do you play computer games on campus? CLASS B
8. FGOSB: How often do you play computer games outside school? CLASS B
9. LGB: Do you like playing computer games? CLASS B
10. EGB: Do you have any experience in playing computer games? CLASS B
11. postDGBI1: Item 1 in the posttest of CGBI
12. postDGBI2: Item 2 in the posttest of CGBI
13. postDGBI3: Item 3 in the posttest of CGBI
14. postDGBI4: Item 4 in the posttest of CGBI
15. postDGBI5: Item 5 in the posttest of CGBI
16. postDGBI6: Item 6 in the posttest of CGBI
17. postDGBI7: Item 7 in the posttest of CGBI
18. postDGBI8: Item 8 in the posttest of CGBI
19. postDGBI9: Item 9 in the posttest of CGBI
20. postDGBI10: Item 10 in the posttest of CGBI
21. postDGBI11: Item 11 in the posttest of CGBI
22. postDGBI12: Item 12 in the posttest of CGBI
23. postDGBI13: Item 13 in the posttest of CGBI
24. postDGBI14: Item 14 in the posttest of CGBI
25. postDGBI15: Item 15 in the posttest of CGBI
26. postDGBI16: Item 16 in the posttest of CGBI
27. postDGBI17: Item 17 in the posttest of CGBI
28. postDGBI18: Item 18 in the posttest of CGBI
29. postDGBI19: Item 19 in the posttest of CGBI
30. postDGBI20: Item 20 in the posttest of CGBI
31. postDGBI21: Item 21 in the posttest of CGBI
32. postDGBI22: Item 22 in the posttest of CGBI
33. postDGBI23: Item 23 in the posttest of CGBI
34. postDGBI24: Item 24 in the posttest of CGBI
35. postDGBI25: Item 25 in the posttest of CGBI
36. postDGBI26: Item 26 in the posttest of CGBI
37. postDGBI27: Item 27 in the posttest of CGBI
38. postDGBI28: Item 28 in the posttest of CGBI
39. postDGBI29: Item 29 in the posttest of CGBI
40. postDGBI30: Item 30 in the posttest of CGBI
41. postND1: Item 1 in the posttest of NCGBI
42. postND2: Item 2 in the posttest of NCGBI
43. postND3: Item 3 in the posttest of NCGBI
44. postND4: Item 4 in the posttest of NCGBI
45. postND5: Item 5 in the posttest of NCGBI
46. postND6: Item 6 in the posttest of NCGBI
47. postND7: Item 7 in the posttest of NCGBI
48. postND8: Item 8 in the posttest of NCGBI
49. postND9: Item 9 in the posttest of NCGBI
50. postND10: Item 10 in the posttest of NCGBI
51. postND11: Item 11 in the posttest of NCGBI
52. postND12: Item 12 in the posttest of NCGBI
53. postND13: Item 13 in the posttest of NCGBI
54. postND14: Item 14 in the posttest of NCGBI
55. postND15: Item 15 in the posttest of NCGBI
56. postND16: Item 16 in the posttest of NCGBI
57. postND17: Item 17 in the posttest of NCGBI
58. postND18: Item 18 in the posttest of NCGBI
59. postND19: Item 19 in the posttest of NCGBI
60. postND20: Item 20 in the posttest of NCGBI
61. postND21: Item 21 in the posttest of NCGBI
62. postND22: Item 22 in the posttest of NCGBI
63. postND23: Item 23 in the posttest of NCGBI
64. postND24: Item 24 in the posttest of NCGBI
65. postND25: Item 25 in the posttest of NCGBI
66. postND26: Item 26 in the posttest of NCGBI
67. postND27: Item 27 in the posttest of NCGBI
68. postND28: Item 28 in the posttest of NCGBI
69. postND29: Item 29 in the posttest of NCGBI
70. postND30: Item 30 in the posttest of NCGBI
71. preD1: Item 1 in the pretest of CGBI
72. preD2: Item 2 in the pretest of CGBI
73. preD3: Item 3 in the pretest of CGBI
74. preD4: Item 4 in the pretest of CGBI
75. preD5: Item 5 in the pretest of CGBI
76. preD6: Item 6 in the pretest of CGBI
77. preD7: Item 7 in the pretest of CGBI
78. preD8: Item 8 in the pretest of CGBI
79. preD9: Item 9 in the pretest of CGBI
80. preD10: Item 10 in the pretest of CGBI
81. preD11: Item 11 in the pretest of CGBI
82. preD12: Item 12 in the pretest of CGBI
83. preD13: Item 13 in the pretest of CGBI
84. preD14: Item 14 in the pretest of CGBI
85. preD15: Item 15 in the pretest of CGBI
86. preD16: Item 16 in the pretest of CGBI
87. preD17: Item 17 in the pretest of CGBI
88. preD18: Item 18 in the pretest of CGBI
89. preD19: Item 19 in the pretest of CGBI
90. preD20: Item 20 in the pretest of CGBI
91. preD21: Item 21 in the pretest of CGBI
92. preD22: Item 22 in the pretest of CGBI
93. preD23: Item 23 in the pretest of CGBI
94. preD24: Item 24 in the pretest of CGBI
95. preD25: Item 25 in the pretest of CGBI
96. preD26: Item 26 in the pretest of CGBI
97. preD27: Item 27 in the pretest of CGBI
98. preD28: Item 28 in the pretest of CGBI
99. preD29: Item 29 in the pretest of CGBI
100. preD30: Item 30 in the pretest of CGBI
101. preN1: Item 1 in the pretest of NCGBI
102. preN2: Item 2 in the pretest of NCGBI
103. preN3: Item 3 in the pretest of NCGBI
104. preN4: Item 4 in the pretest of NCGBI
105. preN5: Item 5 in the pretest of NCGBI
106. preN6: Item 6 in the pretest of NCGBI
107. preN7: Item 7 in the pretest of NCGBI
108. preN8: Item 8 in the pretest of NCGBI
109. preN9: Item 9 in the pretest of NCGBI
110. preN10: Item 10 in the pretest of NCGBI
111. preN11: Item 11 in the pretest of NCGBI
112. preN12: Item 12 in the pretest of NCGBI
113. preN13: Item 13 in the pretest of NCGBI
114. preN14: Item 14 in the pretest of NCGBI
115. preN15: Item 15 in the pretest of NCGBI
116. preN16: Item 16 in the pretest of NCGBI
117. preN17: Item 17 in the pretest of NCGBI
118. preN18: Item 18 in the pretest of NCGBI
119. preN19: Item 19 in the pretest of NCGBI
120. preN20: Item 20 in the pretest of NCGBI
121. preN21: Item 21 in the pretest of NCGBI
122. preN22: Item 22 in the pretest of NCGBI
123. preN23: Item 23 in the pretest of NCGBI
124. preN24: Item 24 in the pretest of NCGBI
125. preN25: Item 25 in the pretest of NCGBI
126. preN26: Item 26 in the pretest of NCGBI
127. preN27: Item 27 in the pretest of NCGBI
128. preN28: Item 28 in the pretest of NCGBI
129. preN29: Item 29 in the pretest of NCGBI
130. preN30: Item 30 in the pretest of NCGBI
131. FQ1: Feedback questionnaire question 1 OVERALL APPEAL DGBI
132. FQ2: Feedback questionnaire question 2 OVERALL APPEAL CGBI
133. FQ3: Feedback questionnaire question 3 OVERALL APPEAL CGBI
134. FQ4: Feedback questionnaire question 4 AVAILABILITY OF LEARNING MATERIAL CGBI
135. FQ5: Feedback questionnaire question 5 AVAILABILITY OF LEARNING MATERIAL CGBI
136. FQ6: Feedback questionnaire question 6 AVAILABILITY OF LEARNING MATERIAL CGBI
137. FQ7: Feedback questionnaire question 7 EDUCATIONAL VALUE CGBI
138. FQ8: Feedback questionnaire question 8 EDUCATIONAL VALUE CGBI
139. FQ9: Feedback questionnaire question 9 EDUCATIONAL VALUE CGBI
140. FQ1N: Feedback questionnaire question 1 OVERALL APPEAL NCGBI
141. FQ2N: Feedback questionnaire question 2 OVERALL APPEAL NCGBI
142. FQ3N: Feedback questionnaire question 3 OVERALL APPEAL NCGBI
143. FQ4N: Feedback questionnaire question 4 AVAILABILITY OF LEARNING MATERIAL NCGBI
144. FQ5N: Feedback questionnaire question 5 AVAILABILITY OF LEARNING MATERIAL NCGBI
145. FQ6N: Feedback questionnaire question 6 AVAILABILITY OF LEARNING MATERIAL NCGBI
146. FQ7N: Feedback questionnaire question 7 EDUCATIONAL VALUE NCGBI
147. FQ8N: Feedback questionnaire question 8 EDUCATIONAL VALUE NCGBI
148. FQ9N: Feedback questionnaire question 9 EDUCATIONAL VALUE NCGBI
149. PostCGBI: Posttest of CGBI
150. PostNCGBI: Posttest of NCGBI
151. PreCGBI: Pretest of CGBI
152. PreNCGBI: Pretest of NCGBI
153. appealCGBI: Appellation of CGBI
154. appealNDGBI: Appellation of NCGBI
155. availCGBI: Availability of learning materials of CGBI
156. availNCGBI: Availability of learning materials of NCGBI
157. eduvalCGBI: Educational value of CGBI
158. eduvalNCGBI: Educational value of NCGBI
159. motCGBI: Motivation of CGBI
160. motNCGBI: Motivation of NCGBI
